# Soybean Crops Penalize Subsequent Wheat Yield During Drought in the North China Plain

**DOI:** 10.3389/fpls.2022.947132

**Published:** 2022-06-28

**Authors:** Jiangwen Nie, Jie Zhou, Jie Zhao, Xiquan Wang, Ke Liu, Peixin Wang, Shang Wang, Lei Yang, Huadong Zang, Matthew Tom Harrison, Yadong Yang, Zhaohai Zeng

**Affiliations:** ^1^College of Agronomy and Biotechnology, China Agricultural University, Beijing, China; ^2^Institute of Agricultural Resources and Regional Planning, Chinese Academy of Agricultural Sciences, Beijing, China; ^3^Tasmanian Institute of Agriculture, University of Tasmania, Launceston, TAS, Australia; ^4^Department of Soil and Plant Microbiome, Institute of Phytopathology, Christian-Albrechts-University of Kiel, Kiel, Germany

**Keywords:** pre-crop effect, water deficient, cropping system, wheat yield, nitrogen fertilization, drought

## Abstract

Contemporary wisdom suggests that inclusion of legumes into crop rotations benefit subsequent cereal crop yields. To investigate whether this maxim was generically scalable, we contrast summer soybean–winter wheat (SW) with summer maize–winter wheat (MW) rotation systems in an extensive field campaign in the North China Plain (NCP). We identify heretofore unseen interactions between crop rotation, synthetic N fertilizer application, and stored soil water. In the year with typical rainfall, inclusion of soybean within rotation had no effect on wheat ear number and yield, while N fertilization penalized wheat yields by 6–8%, mainly due to lower dry matter accumulation after anthesis. In contrast, in dry years prior crops of soybean reduced the rate and number of effective ears in wheat by 5–27 and 14–17%, respectively, leading to 7–23% reduction in wheat yield. Although N fertilization increased the stem number before anthesis in dry years, there was no corresponding increase in ear number and yield of wheat in such years, indicating compensating reduction in yield components. We also showed that N fertilization increased wheat yield in MW rather than SW as the former better facilitated higher dry matter accumulation after flowering in dry years. Taken together, our results suggest that soybean inclusion reduced soil available water for subsequent wheat growth, causing yield penalty of subsequent wheat under drought conditions. We call for more research into factors influencing crop soil water, including initial state, crop water requirement, and seasonal climate forecasts, when considering legumes into rotation systems.

**Graphical Abstract:**
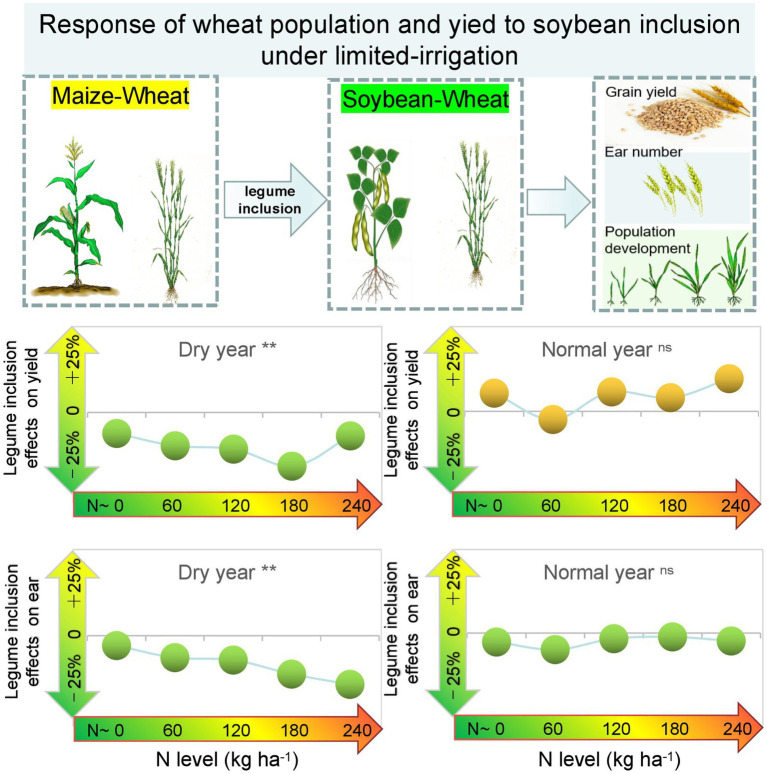
Response of wheat population and yield to soybean inclusion under limited-irrigation.

Response of wheat population and yield to soybean inclusion under limited-irrigation.

## Highlights

– Soybean inclusion decreased soil water storage for subsequent wheat.
– Precipitation years affected the pre-crop and N fertilization impacts on wheat population and yield.
– Soybean inclusion reduced wheat yield and ear number in dry years.
– N fertilization reduced dry matter accumulation after flowering and yield of wheat in normal years.

## Introduction

Optimizing wheat population is crucial to increasing chances of attaining potential wheat grain yield, in which optimal population is a function of population density and quality with balanced yield components (Xu et al., 2013). In China, optimizing the wheat population canopy and coordinating the development between wheat population quantity and quality may be conducive to higher yields of wheat (≥9 t ha^−1^) under appropriate management practices (Meng et al., 2013). In the North China Plain (NCP), farmers often apply excessive irrigation or nitrogen (N) fertilizer in attempt to maximize wheat growth. However, these unreasonable practices usually result in sub-optimal population, reflected by excessive population density and high tiller mortality, thus reducing wheat yield (Li et al., 2012). As well, excessive use, inappropriate timing, and seasonal conditions as well as form of N fertilizer may contribute to either excessive N leaching into ground water and eutrophication, supra-optimal N volatilization, or extreme pulses of greenhouse gas emissions (Harrison et al., 2014; Christie et al., 2018; Smith et al., 2021). Collectively, these factors suggest that better understanding the dynamics and driving factors of population establishment will be vital to achieving ensuring agri-food system intensification without causing adverse effects of natural resources and the environment (Harrison et al., 2021; Yan et al., 2022).

In general, yield reduction in winter wheat may be due to either low productive tiller production before and during winter (Meng et al., 2013), or low dry matter accumulation after flowering since unproductive tillers in spring compete for water and nutrients with productive tillers (Peng et al., 2008). First, while tiller number determines canopy densities during early development, not all tillers survive to maturation (Davidson and Chevalier, 1990). The rate of the productive tiller development is regulated by manifold environmental factors, including soil temperature (Thorne and Wood, 1987; Liu et al., 2022), soil water (Tao et al., 2007), soil nutrient supply (Longnecker et al., 1993; Scharf and Alley, 1993), and defoliation (Harrison et al., 2012). For instance, Shang et al. (2020) reported that adequate water supply at jointing stage can effectively reduce the tiller mortality and thus increase wheat yield. On the other hand, kernel assimilate is primarily derived from two temporal periods: the transfer of photosynthetic assimilate stored in vegetative organs before flowering (this is used to build ear organs); and the other comes from the accumulation of stored assimilates plus photosynthesis after flowering, which is used for grain filling (Harrison et al., 2012). Consequently, the core indicator of wheat population quality is canopy photosynthetic production from flowering to maturity (Ma et al., 2020; Wang et al., 2020). Correspondingly, decreased dry matter on individual plants may result in resistance weak and plant mortality (Sui et al., 2013). Optimal crop management is thus essential for establishing ideal populations to enhance productive tiller development and simultaneously limit unproductive growth.

In addition to the selection of cultivars with different genetic tillering propensities, N application is considered to be an effective practice to stimulate tillering and regulate population development (Weisz et al., 2001; Engström and Bergkvist, 2009; Zhang et al., 2020). In attempt for greater yields, farmers often apply excessive N fertilizer at sowing to increase the number of ears (spikes; Chen et al., 2011). Nitrogen fertilizer responses and crop development may impact on flowering time, which can also determine whether potential yields are attained or not (Liu et al., 2020a). However, this can result in larger, heavier canopies that can lodge in later development (Bond et al., 2008). Higher N application rate may also reduce N use efficiency (Wang et al., 2014), increase ammonia volatilization (Smith et al., 2021) and impede the sustainable development of wheat production in the NCP (Li et al., 2017; Huang et al., 2018). There is thus an urgent need for development of management practices that optimize N fertilization management across systems (Rawnsley et al., 2019), but particularly in summer maize−winter wheat (MW) rotation system to maintain high yield but improve environmental sustainability in the NCP.

Incorporation of legumes within cereal crop rotations is believed to reduce the need for input for N fertilizers and optimize yield, which mainly provides nutrients for the next crop of wheat through N fixation of legume crops (Oberson et al., 2013; Amossé et al., 2014; Manevski et al., 2015; Peoples et al., 2017; Plaza-Bonilla et al., 2017). In addition to the “N-effects,” the observed benefits of grain legumes on subsequent crops also include “non-N-effects,” which is divided into biotic (occurrence of pests, weeds, and diseases) and abiotic factors (availability of water or nutrients except for N in soil). However, performance of crops within legume-cereal rotations is affected by seasonal climate conditions and soil fertility (Bell et al., 2015). For example, Franke et al. (2018) reported that synthetic N application to cereals reduces the residual effects of legumes, but the response at low N (60–120 kg N ha^−1^) input is still positive compared with no-N management. Nevertheless, other studies demonstrated that legume inclusion reduced soil water storage due to higher leaf area index (Qin et al., 2018; Zhang et al., 2021; Nie et al., 2022), which may cause water shortage in the wheat-growing season. Therefore, the wheat population and yield in legume-cereal rotation systems with different N managements needs to be further investigated, especially under water-deficit and drought conditions. Such studies should help disentangle the interplay between (1) N use, (2) biomass production, (3) soil water use of legumes, and (4) seasonal climatic conditions on subsequent crop development.

The NCP produces approximately 60% of national wheat and plays a pivotal role in national food security (National Bureau of Statistics, 2020). In recent years, summer soybean−winter wheat (SW) rotation system is considered as a feasible alternative to MW in the NCP, due to higher potentials to reduce environmental costs without sacrificing crop yield (Mupangwa et al., 2021). In addition, limited-irrigation (applied at sowing and jointing) has been widely used for winter wheat in the NCP due to the groundwater depletion (Xu et al., 2016; Wang et al., 2020). However, there is yet to be optimized for SW rotation system under limited irrigation. Therefore, the aims of our study were: (i) to quantify grain yield of legume crop introduction (i.e., soybean) as part of crop rotation under different N management practices, and (ii) to quantify the crop population response to rotation and N management practices.

## Materials and Methods

### Site Description

A 3-year (June 2018 to June 2021) field experiment was conducted in the Wuqiao Experimental Station of China Agricultural University (37°41′N, 116°36′E), Cangzhou City, Hebei, China. This region has a typical sub-humid continental monsoon climate with cold winter and hot summer. The annual active accumulated temperature (≥0°C) and the annual frost-free period are 4,826°C and 201 days, respectively (Zhang et al., 2019; Zhao et al., 2019). The long-term average annual rainfall is 562 mm, with a characteristic of erratic seasonal distribution and a sharp yearly fluctuation (Wang et al., 2021). The soil had a Calcaric Fluvisol developed on an alluvial plain texture with a soil pH (H_2_O) of 7.74, soil organic carbon (SOC) of 9.0 g kg^−1^, total nitrogen (TN) of 1.3 g kg^−1^, total phosphorus (TP) of 1.73 g kg^−1^, and available phosphorous (Olsen-P) of 89.8 mg kg^−1^ (Wang et al., 2021).

### Experimental Design and Crop Management

The field experiment was arranged in a split-plot design with rotation system as the main plot and nitrogen (N) treatments as subplot. The plot size was 60 m^2^ (6 × 10 m) with three field replicates. Two rotation systems: summer soybean−winter wheat (SW) and summer maize−winter wheat (MW) were ranged in the main plot. The five N treatments were: 0 kg N ha^−1^ (N0), 60 kg N ha^−1^ (N1), 120 kg N ha^−1^ (N2), 180 kg N ha^−1^ (N3), and 240 kg N ha^−1^ (N4) for winter wheat.

Before sowing, summer crops (soybean and maize) were surface irrigated at 750 m^3^ ha^−1^; and after sowing, basal fertilizer for summer crops were applied with the combination of designed N application rate (summer soybean and maize were applied with 60 and 120 kg N ha^−1^, respectively) and 103.5 kg ha^−1^ of P_2_O_5_ and 112.5 kg ha^−1^ of K_2_O. Summer maize (*cv.* Zhengdan 958) and soybean (*cv*. Xudou 20) were sown in Mid-June with a row spacing of 0.6 and 0.4 m, and a plant spacing of 0.24 and 0.15 m, respectively, and they were both harvested in Early-October from 2018 to 2021.

Before winter wheat sowing, straw of maize and soybean was incorporated. In NCP, farmers often applied all fertilizer as basal due to lower labor (Xu et al., 2016; Wang et al., 2020), thus the basal fertilizer was applied with the combination of designed N application rate and 138 kg ha^−1^ of P_2_O_5_ and 112.5 kg ha^−1^ of K_2_O. The form of chemical fertilizers was applied as the urea, diammonium phosphate, and potassium sulfate. Winter wheat (*cv*. Jimai 22) with 15 cm rows space was sown at Mid-October with a sowing rate of 337.5, 315, and 300 kg ha^−1^ in 2018/2019, 2019/2020, and 2020/2021 seasons, respectively. Wheat was harvested on June 3, 2019, June 8, 2020, and June 3, 2021, respectively. Winter wheat was surface irrigated before wheat sowing and fertilizer applied (750 m^3^ ha^−1^) and again at the jointing stage (750 m^3^ ha^−1^).

### Sampling and Measurement

The stem number was counted in two 1-m row sites at tillering (3 weeks after sowing), overwintering (7–8 weeks after sowing), jointing (22–23 weeks after sowing), and anthesis (28–29 weeks after sowing), respectively. The rate of effective ear was calculated as the ratio of ear number at harvest to stem number at anthesis (Lu et al., 2014). The ear (panicle) number and grain number per ear were determined in two sites with 1 m^2^ and the grains of each ear from 40 randomly selected wheat plants at harvest, respectively.

Wheat grain yield (GY), aboveground biomass (AGB), and harvest index (HI) were measured based on the average of two sites with 2 m^2^ (1 m × 1 m). The grain yield was reported at 14% moisture. Harvest index is calculated as the ratio of grain yield to AGB at wheat maturity (Wang et al., 2021):


$$HI=GY/AGB\;\left(1\right)$$

The dry matter accumulation was sampled from 0.3 m^2^ in each plot. Then, wheat plant samples were oven-dried at 75°C until achieving constant dry weight. Post-anthesis dry matter accumulation (DMpa) was determined by the dry matter accumulation difference between maturity and flowering stage of wheat (Wang et al., 2021):


$$\mathrm{DMpa}=AGB-DMa\;\left(2\right)$$

The contribution of DMpa to wheat grain yield was determined by the ratio of DMpa to grain yield at maturity (CR).


$$CR\left(\%\right)=\mathrm{DMpa}/GY\times 100\;\left(3\right)$$

Soil water content was determined at summer crops and wheat harvest (Early-October) and wheat sowing (Mid-October). Soil samples were taken from 0 to 2 m with 20-cm intervals by a ground auger, then dried at 105°C to a constant weight. The soil water storage (SWS, mm) of each layer was calculated as follows (Wang et al., 2021):


$$SWS=SWC\times D\times SD\;\left(4\right)$$

Where SWC (%) is the water content of dry soil, *D* (g cm^−3^) is the soil bulk density, and SD (mm) is soil depth.

### Data Analysis

The Data Processing System (DPS) software version 7.05 was performed for statistical analyses (Tang and Zhang, 2013). A three-way ANOVA was used to show the effects of experimental year, rotation system, and N fertilization rate on grain yield and yield components of winter wheat with Fisher’s Least Significant Difference (LSD) test (*p* < 0.05). We checked the residuals to ensure normality and homogeneity by Shapiro and Leneve’s tests, respectively. When the normality test fails, the data transform was performed by log. SigmaPlot version 12.5 (Systat Software Inc., San Jose, CA, United States) was used to perform the Figures.

## Results

### Weather Conditions and Soil Water Storage Before Winter Wheat Sowing

**Figure 1:**
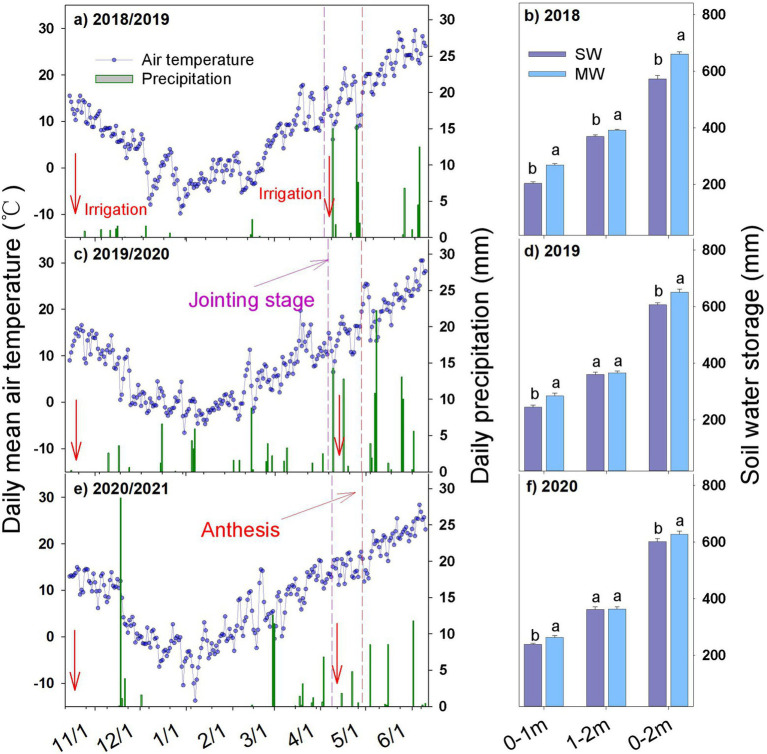
Weather condition **(A,C,E)** during winter wheat growing seasons and soil water storage **(B,D,F)** before winter wheat sowing in summer maize−winter wheat (MW) and summer soybean−winter wheat (SW) rotation systems. Values are means + SEs (*n* = 3) for soil water storage. Different lowercase letters indicate significant differences (*p* < 0.05).

Daily average temperature in 2019/2020 season (8.58°C) was higher by 0.64 and 0.43°C than those in 2018/2019 and 2020/2021 seasons, respectively (Figures 1A,C,E). Daily precipitation varied significantly across seasons. The precipitation accumulation was 48.7, 153.6, and 109.8 mm in the 2018/2019, 2019/2020, and 2020/2021 seasons, respectively. According to the amount of precipitation, the 3 years were divided into wet, normal, and dry years based on the empirical frequency analysis (Zhao et al., 2020; Gao et al., 2022). Therefore, 2018/2019 season was a typical dry season, while 2020/2021 and 2019/2020 seasons were normal seasons. However, 66% of precipitation (73.4 mm) occurred before the wheat jointing stage in 2020/2021 (Figure 1E), thus 2020/2021 season was a dry season.

The soil water storage (SWS) in the 0–2 m soil depth before wheat sowing differed significantly between rotation systems in the 3 years (Figures 1B,D,F; Supplementary Figure S1). The SWS of SW in the 0–1, 1–2, and 0–2 m soil depths was 64.6, 22.5, and 87.1 mm and 39.6, 5.0, and 44.6 mm and 24.5, 1.5, and 26 mm lower than those of MW in 2018/2019, 2019/2020, and 2020/2021 seasons, respectively (Figures 1B,D,F). Moreover, SW depleted more soil water at the surface layer (0–60 cm, Supplementary Figure S1) in summer, but reduced soil water consumption in the deeper soil layer (> 60 cm) over 3 years, as compared with MW.

### Dynamics of Wheat Stem in SW and MW Under Five N Application Rates

**Figure 2:**
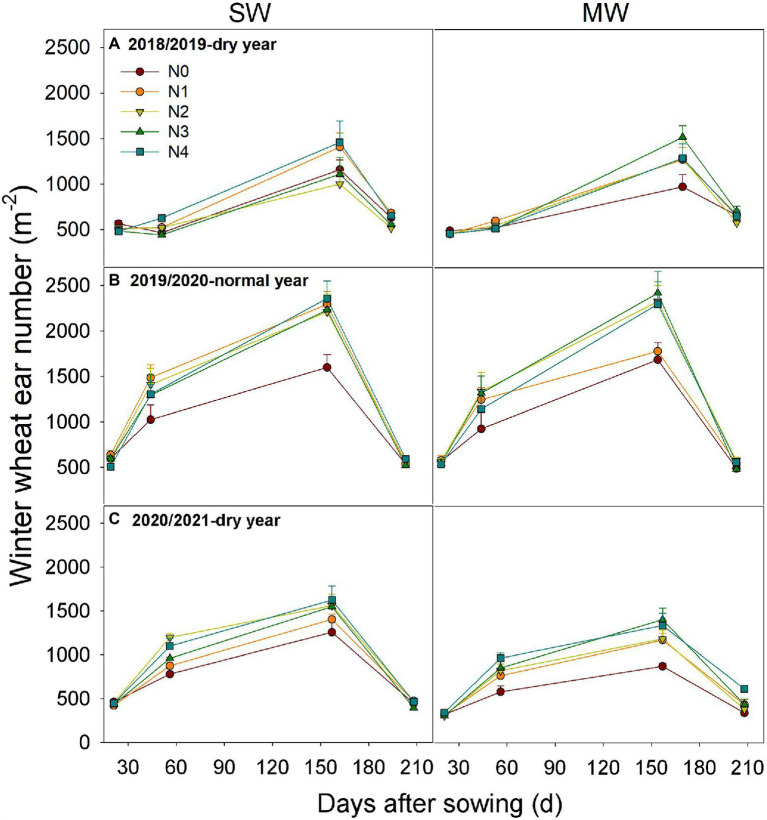
Dynamics of wheat ear number in summer soybean−winter wheat (SW) and summer maize−winter wheat (MW) rotation systems under five N application rates. Values are means + SEs (*n* = 3). N0, 0 kg N ha^−1^; N1, 60 kg N ha^−1^; N2, 120 kg N ha^−1^; N3, 180 kg N ha^−1^; and N4, 240 kg N ha^−1^.

At the jointing stage of winter wheat, the stem number reached highest but then quickly declined until the anthesis stage (Figure 2). In both rotation systems, more stems were recorded under higher N application rates than those under lower application rates from the overwinter to anthesis stage. However, a minor difference in stem number was found between SW and MW under the same N application rate.

The rate of effective ear of SW were 4.8 and 27.4% lower than those of MW across five N treatments in dry years (2018/2019 and 2020/2021), respectively, whereas no significant difference was observed in normal year (2019/2020, Figure 3). Moreover, N application reduced the rate of effective ear in all 3 years across rotation systems as compared with N0. Overall, rotation system had a minor effect on the dynamic of stem number during the early stage of wheat under the same N application rate, but resulted in a lower effective ear number at wheat harvest.

**Figure 3:**
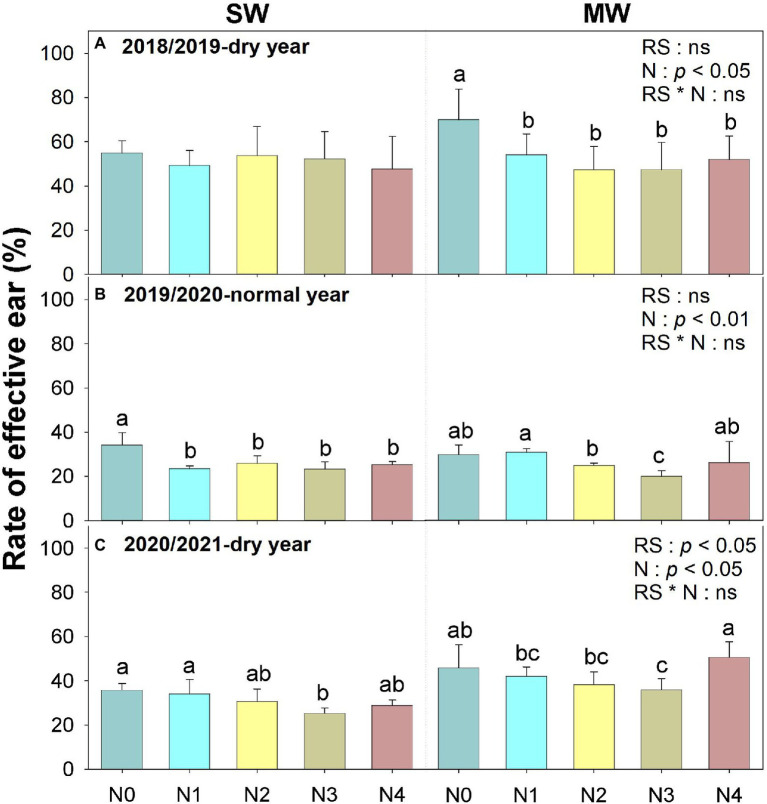
The rate of effective ear of winter wheat in summer soybean–winter wheat (SW) and summer maize–winter wheat (MW) rotation systems under five N application rates. Values are means + SEs (*n* = 3). Different lowercase letters indicate significant differences (*p* < 0.05) among five fertilization treatments within the same rotation system in the same year. RS, rotation system; N, nitrogen fertilization; and CS × N, interaction of rotation system and nitrogen fertilization. N0, 0 kg N ha^−1^; N1, 60 kg N ha^−1^; N2, 120 kg N ha^−1^; N3, 180 kg N ha^−1^; and N4, 240 kg N ha^−1^.

### Dry Matter Accumulation of Wheat in SW and MW Under Five N Application Rates

The aboveground biomass (AGB) in normal year was 16.7 Mg ha^−1^, which was 25 and 31% higher than those in dry years (*p* < 0.01; Tables 1, 2), respectively. Legume inclusion reduced AGB by 16 and 19% in dry years, as compared with MW. The AGB increased with the increased N application rates regardless of rotation systems.

**Table 1:** Combined analysis of variances for grain yield, agronomical and physiological traits of winter wheat.

ANOVA	Year (Y)	Rotation system (R)	Nitrogen fertilization (N)	Y × R	Y × N	R × N	Y × R × N
Grain yield	**	**	ns	**	ns	ns	ns
Ear number	**	**	ns	ns	ns	ns	ns
Rate of effective ear	*	**	*	*	ns	ns	ns
Grain number	**	ns	ns	ns	ns	ns	ns
TGW	**	ns	**	ns	**	ns	ns
AGB	**	*	**	ns	*	**	*
HI	**	ns	*	ns	ns	*	ns
DMa	*	*	**	ns	ns	*	ns
DMpa	*	ns	ns	ns	**	**	*
CR	*	ns	ns	ns	ns	**	ns

* *p* *< 0.05;*

** *p < 0.01*.

*ns, not significant difference*.

*TGW, thousand grain weight; AGB, aboveground biomass; HI, harvest index. DMa, dry matter accumulation at anthesis. DMpa, dry matter accumulation during post-anthesis. CR, contribution ratio of DMpa to grain yield*.

**Table 2:** Yield components, dry matter accumulation at anthesis (DMa), dry matter accumulation during post-anthesis (DMpa), and contribution ratio of DMpa to grain yield (CR) of winter wheat in summer soybean−winter wheat (SW) and summer maize−winter wheat (MW) rotation systems under five N application rates.

Rotation system	Nitrogen fertilization	Ear number (m^−2^)	Grain number (ear^−1^)	TGW (g)	AGB (Mg ha^−1^)	HI	DMa (Mg ha^−1^)	DMpa (Mg ha^−1^)	CR (%)
2018/2019									
SW	N0	573.8ab	29.3a	40.3a	10.6bc	0.46ab	6.1c	4.5ab	42.2a
	N1	597.0a	30.1a	38.8a	13.3a	0.43bc	6.9bc	6.4a	47.5a
	N2	593.5a	30.4a	38.9a	9.7c	0.54a	7.9ab	1.8c	18.1b
	N3	546.0b	28.5a	39.5a	12.7a	0.37c	6.3c	6.5a	50.9a
	N4	557.7ab	30.9a	37.7a	11.3ab	0.44abc	8.5a	2.8bc	24.4b
	**Mean**	**573.6B**	**29.8A**	**39.0A**	**11.5A**	**0.49A**	**7.1B**	**4.4A**	**36.6A**
MW	N0	646.3b	35.5a	41.6a	12.5b	0.53a	7.9c	4.6a	36.5a
	N1	640.7b	35.0a	40.2ab	13.6ab	0.47a	8.1bc	5.5a	40.3a
	N2	687.5ab	35.4a	38.5b	13.9ab	0.47a	8.3bc	5.6a	39.9a
	N3	650.0b	33.5a	38.7b	13.5ab	0.51a	9.9a	3.6a	26.4a
	N4	723.0a	36.5a	38.8b	14.6a	0.47a	9.4ab	5.2a	32.8a
	**Mean**	**669.5A**	**35.2A**	**39.6A**	**13.6A**	**0.45A**	**8.7A**	**4.9A**	**35.2A**
2019/2020									
SW	N0	779.5a	30.8a	41.9a	18.5a	0.43b	6.3b	12.2a	65.4a
	N1	811.5a	31.9a	40.2bc	14.9b	0.50a	7.4a	7.5b	49.4b
	N2	833.5a	32.1a	41.3a	16.5ab	0.45ab	8.0a	8.6b	51.4b
	N3	852.5a	31.0a	39.7c	16.2ab	0.43ab	8.2a	8.0b	49.4b
	N4	832.0a	33.1a	40.9ab	18.4a	0.42b	7.0ab	11.4a	61.3a
	**Mean**	**821.8A**	**31.8A**	**40.8A**	**16.9A**	**0.45A**	**7.4A**	**9.5A**	**55.4A**
MW	N0	817.5a	32.8a	41.0a	15.6ab	0.48a	6.0b	9.6ab	61.9a
	N1	888.0a	29.5a	40.7a	17.7ab	0.44a	7.8a	9.9a	55.6ab
	N2	858.3a	29.6a	38.6c	15.7ab	0.43a	7.5a	8.2ab	52.0bc
	N3	869.5a	30.0a	39.4bc	15.5b	0.43a	8.1a	7.4b	47.4c
	N4	867.5a	32.6a	39.9ab	18.2a	0.37b	8.6a	9.6ab	52.7bc
	**Mean**	**860.2A**	**30.9A**	**39.9A**	**16.5A**	**0.43A**	**7.6A**	**8.9A**	**53.9A**
2020/2021									
SW	N0	457.7ab	38.7a	46.0b	9.3b	0.43a	5.6ab	3.8b	39.9b
	N1	424.2ab	37.2a	48.0a	8.8b	0.45a	5.4ab	3.4b	38.2b
	N2	486.7a	38.5a	48.3a	10.7a	0.34c	6.0a	4.7b	43.1ab
	N3	438.5ab	39.5a	47.9a	11.5a	0.36bc	4.7b	6.9a	58.6a
	N4	406.0b	40.2a	45.0b	10.9a	0.42ab	6.3a	4.5b	41.1b
	**Mean**	**442.6B**	**38.8A**	**47.0A**	**10.3A**	**0.40A**	**5.6A**	**6.8A**	**44.2A**
MW	N0	454.2c	37.9b	44.8ab	9.4c	0.41a	4.2b	5.2c	56.3a
	N1	507.3bc	38.3b	46.0a	13.1b	0.36ab	5.6a	7.5ab	56.4a
	N2	553.7ab	39.6ab	44.2ab	13.1b	0.32bc	6.2a	6.9abc	52.3a
	N3	581.8a	39.5ab	43.7b	12.0b	0.40a	6.4a	5.6bc	46.6a
	N4	568.8ab	41.9a	42.0c	16.0a	0.27c	7.1a	8.9a	56.2a
	**Mean**	**533.2A**	**39.4A**	**44.1B**	**12.7A**	**0.35A**	**5.9A**	**4.7A**	**53.5A**

*Values are means (*n* = 3). Different lowercase letters indicate significant differences (*p* < 0.05) among five fertilization treatments within the same rotation system in the same year. Different uppercase letters indicate significant differences (*p* < 0.05) between summer soybean–winter wheat and summer soybean–winter wheat rotation systems in the same year. TGW, thousand grain weight; AGB, aboveground biomass; HI, harvest index. N0, 0 kg N ha^−1^; N1, 60 kg N ha^−1^; N2, 120 kg N ha^−1^; N3, 180 kg N ha^−1^; and N4, 240 kg N ha^−1^. Bold values represent means of different N fertilization treatments within the same rotation system*.

N application increased DMa in both cropping systems as compared with N0 across the 3 years, whereas legume inclusion decreased DMa in dry years (Table 2). Similar to AGB at maturity, DMa increased with the increasing of N application rate in both rotation systems. However, the dry matter accumulation of wheat after anthesis (DMpa) and contribution ratio of DMpa to grain yield were not affected by rotation system and N fertilization, but affected by the interaction of rotation system and N fertilization (Tables 1, 2).

### Wheat Yield and Yield Components in SW and MW Under Five N Application Rates

**Figure 4:**
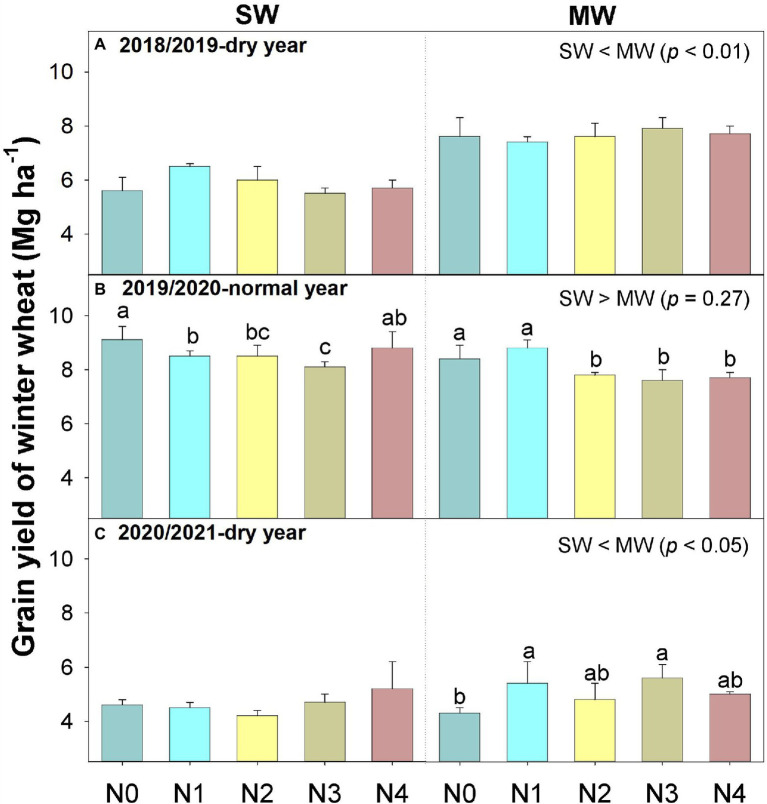
Grain yield of winter wheat in summer soybean–winter wheat (SW) and summer maize–winter wheat (MW) rotation systems under five N application rates. Values are means + SEs (*n* = 3). Different lowercase letters indicate significant differences (*p* < 0.05) among five fertilization treatments within the same rotation system in the same year. N0, 0 kg N ha^−1^; N1, 60 kg N ha^−1^; N2, 120 kg N ha^−1^; N3, 180 kg N ha^−1^; and N4, 240 kg N ha^−1^.

**Figure 5:**
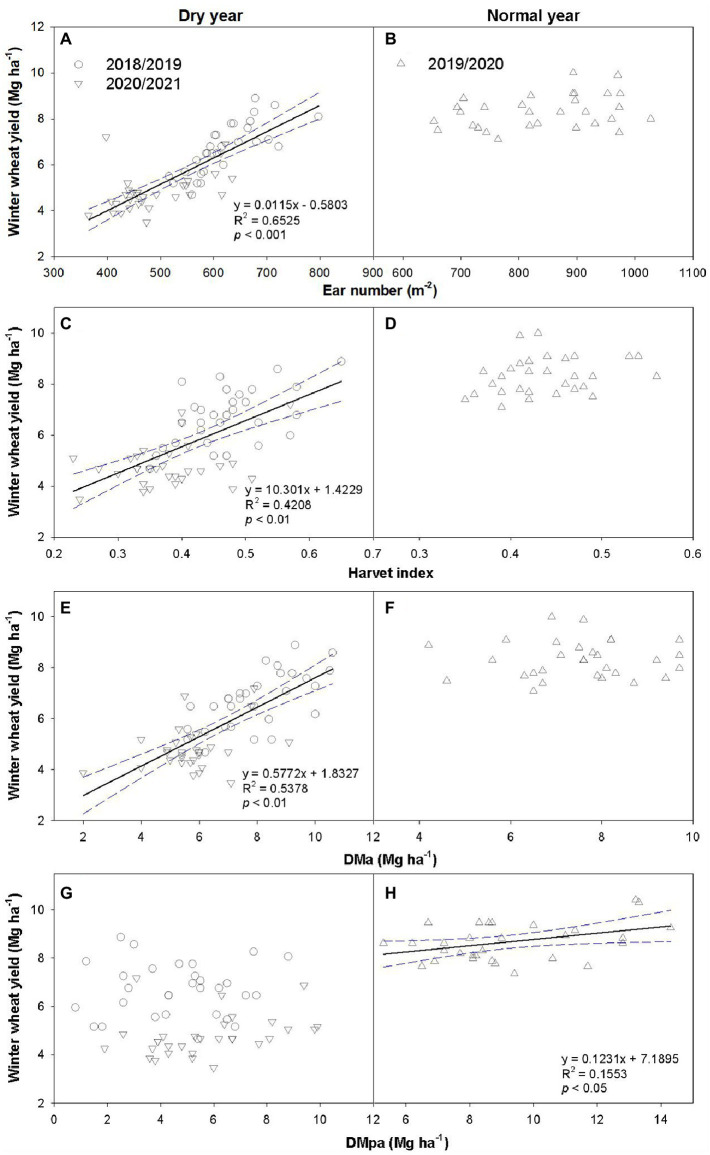
Relationships between wheat yield and ear number **(A,B)**, harvest index **(C,D)**, dry matter accumulation at anthesis (DMa; **E**,**F**), and dry matter accumulation during post-anthesis (DMpa; **G**,**H**) in dry and normal years, respectively.

Wheat yield was significantly influenced by precipitation year, rotation system, and interaction of year and rotation system (*p* < 0.001, Figure 4). Wheat yield in normal year (7.6–9.1 Mg ha^−1^) was 19–42% higher than those in dry years (5.6–7.9 Mg ha^−1^ in 2018/2019 and 4.2–5.6 Mg ha^−1^ in 2020/2021). Moreover, soybean incorporation reduced wheat yield by 23 and 7% in dry years compared with MW, respectively (Figures 4A,C). However, the response of wheat yield to the N rate showed an opposite trend between years. Regardless of rotation systems, N applications had no impact on wheat yield in 2018/2019 season but decreased wheat yield by 3–12% in normal year. In 2020/2021 season, N application increased wheat yield of MW by 10–29%, while no variation was observed of SW as compared with N0. Grain yield is positively related with DMpa in 2019/2020 season, and positively related with DMa in dry years (Figure 5). Overall, the introduction of legumes into rotation exhibited a neutral or negative effect on subsequent wheat yield.

Following trends in yields, ear numbers in dry years were 26 and 42% lower than in normal year, respectively (*p* < 0.01, Table 2). As well, SW reduced wheat ear number by 14 and 17% in the dry years compared with MW, respectively (Table 2). Thousand grain weight (TGW) was influenced by year, N application rate, and their interaction (Table 1), the TGW in MW showed a declining trend with increased N application rates within the 3 years (Table 2). Harvest index (HI) also decreased with increasing N application rate in both cropping systems.

**Figure 6:**
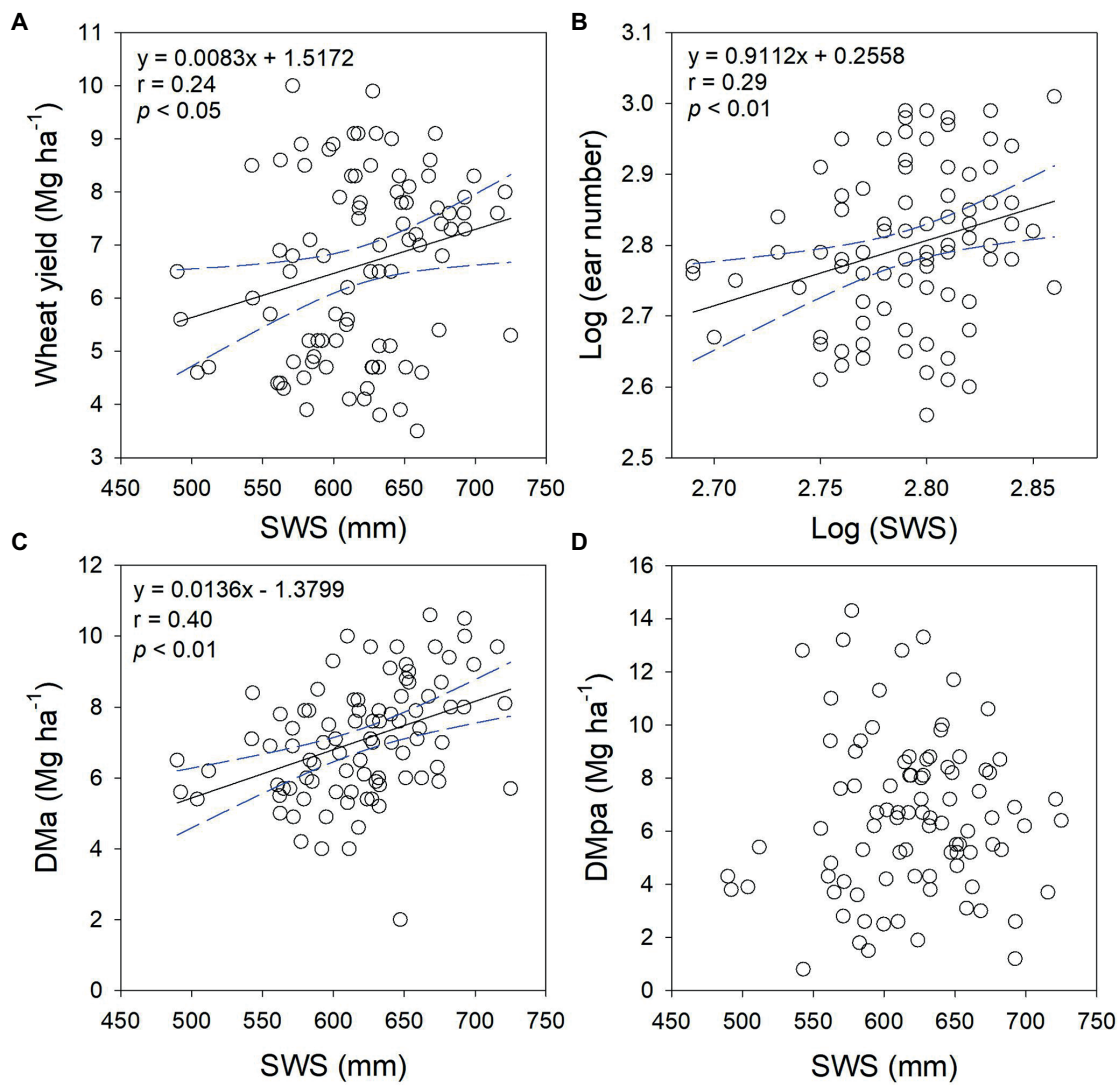
Relationships between wheat yield **(A)**, ear number **(B)**, dry matter accumulation at anthesis (DMa, **C**), and dry matter accumulation during post-anthesis (DMpa, **D**) and soil water storage (SWS) before winter wheat sowing, respectively.

In dry years, ear number was positively correlated with wheat grain yield, regardless of cropping system, N application rate, and experimental year (*p* < 0.01, Figure 5), suggesting that ear number was the main yield component determining wheat yield. Moreover, both AGB and HI positively correlated with wheat grain yield (*p* < 0.01, Figure 5). The SWS significantly and positively related with grain yield, ear number, and dry matter accumulation at anthesis (*p* < 0.01, Figure 6), but not dry matter accumulation during post-anthesis.

## Discussion

### Wheat Population Response to Cropping System and N Application Rate

Optimal N fertilizer management not only matches the N supply with crop N demand (Chen et al., 2006; Cui et al., 2011; Yue et al., 2012), but also ensures adequate N partitioning into productive tillers. Previous studies have shown that during the early tillering period, N application stimulates tiller production (Weisz et al., 2001; Lu et al., 2014) and also promotes the rate of productive stem appearance. Similarly, N application promoted stem numbers during overwintering to anthesis. In contrast to previous studies, we showed that N application did not improve the rate of effective ear correspondingly, but reduced the rate of effective ear to a certain extent (Figures 2, 3). This is mainly because that unproductive tillers promoted by N application compete for light, nutrients, and water with productive tillers (Nuruzzaman et al., 2000; Ao et al., 2010), thus resulting in a lower rate of effective ear. On the other hand, under water-deficient condition, soil water availability affects the critical N dilution curves in crops (Ciampitti et al., 2021). In practice, most farmers usually applied all fertilizer at sowing and limited irrigation at sowing and jointing stages of wheat due to water shortage and high labor costs (Xu et al., 2016). Thus, N application had different impacts on the ear number at wheat harvest between cropping systems and among precipitation years. For example, our results indicated that varying N as basal fertilizer can improve ear number in MW in dry years, but not in SW (Table 2). This may be explained by the fact that ear number was positively correlated with soil water storage (SWS) at sowing (Figure 6). Consequently, the N application as basal fertilizer promotes effective ear number of MW due to higher SWS. Moreover, the variation of ear number was observed among different N application rates in dry years but not normal year, this was mainly attributed to a higher precipitation in normal year than that in dry years (Figure 1). N fertilizer stimulates tillering of wheat during abundant rainfall. However, a higher population (ear number averaged 822–860 m^−2^) in normal year than that in dry years (ear number averaged 442–670 m^−2^) also caused stronger competition for light, water, and nutrition in later wheat growth (Lu et al., 2014). Furthermore, stronger competition resulted in the death of ineffective tillers, thus it had no significant effect on the final number of effective ear. Overall, our results demonstrated that the soil available water and precipitation buffer against the positive effect of N application on wheat tiller dynamic under limited irrigation. Future studies would do well to further dissect the interaction between N and irrigation management in cereal-legume rotations. The interplay between soil water at sowing, within-season precipitation could be explored further using seasonal climate forecasting approaches (Harrison et al., 2017; Liu et al., 2020b) in similar crop rotational systems.

We found that soybean did not impact the stem number of subsequent wheat crops before anthesis when the same N was applied (Figure 2), indicating that legume inclusion did not show an N-benefit for the following wheat under water-deficient conditions. However, soybean inclusion reduced the rate of effective ear as compared with MW, indicating that more productive stem getting senescing in the late growing season. Since the lower soil water storage of SW than that of MW before wheat sowing (Figure 1), it aggravated competition for water and nutrients with productive stems and higher tiller mortality. This was further confirmed by the positive correlation between ear number and soil water storage before wheat sowing (Figures 5, 6). A similar result was reported by Richards et al. (2014), who observed that the low water storage before sowing reduces the number of ears and the rate of effective ear. Thus, legume inclusion induced a reduction in soil water storage for the following wheat (Zhang et al., 2021).

In addition to ear number, dry matter partitioning (before and after anthesis) is one of the main controlling population factors for wheat yield under water-deficit conditions (Wang et al., 2021). Consistent with previous studies (Ehdaie and Waines, 2001; Ma et al., 2020), our results showed that DMa increased with the increase of N application across 3 years, which was attributed to higher nutrition acquisition of tiller under higher N application. However, soybean inclusion reduced DMa in the dry years, which could be explained by the lower SWS of SW before wheat sowing (Richards et al., 2014). In general, many studies believe that DMpa is more important to yield than DMa (Shi et al., 2016), and its contribution to yield reach 38–158% under restricted irrigation conditions (Wang et al., 2021). However, our study indicated that yield depended on DMa in dry years, whereas it mainly depended on DMpa in normal year (Figure 5). This is mainly because the contribution of dry matter accumulation before and after flowering to yield is affected by soil moisture (Foulkes et al., 2007; Thapa et al., 2019). For example, a previous study proved that water deficit at the grain filling stage is detrimental for DMpa, but can significantly increase the transport of DMpa to grain, and thus increase the contribution rate of stem-sheath stored matter to grain yield (Foulkes et al., 2007). Furthermore, the contribution of DMpa to grain yield (CR) in dry years is lower than that in normal year, indicating that precipitation affects CR. Overall, the interannual variation in precipitation leads to the importance of pre- and post-anthesis dry matter accumulation in yield formation.

### Wheat Yield Response to Cropping System and N Application Rate

Increased grain yields are usually obtained when a cereal follows a grain legume in sequence compared with a cereal-cereal rotation. In general, the different rotational effects of grain legumes on subsequent crops could be divided into “N-effects” and “non-N-effects,” and the “non-N-effects” of grain legumes in rotations refers to impacts mediated by biotic factors (the occurrence of pests, weeds, and diseases) and abiotic factors (changes in the availability of water or nutrients other than N; Franke et al., 2018). Similarly, our study found that the “N-effects” and “non-N-effects” of soybean dominated the response of following wheat. In dry years, soybean crops reduced wheat yield, mainly due to lower effective ear numbers resulting from less stored soil water before wheat sowing caused by pre-crop soybean (Supplementary Figure S1; Figure 6). This result is in line with previous studies (Qin et al., 2018; Zhang et al., 2021; Nie et al., 2022). In normal year, although crop rotation did not increase wheat yield, it showed an increasing trend (Figure 4), which was mainly due to increased DMpa to a certain extent (Table 2). These results indicated that the legume inclusion increased soil mineral N for wheat in the year with abundant precipitation. In other words, the impact of legume inclusion on wheat depends on the precipitation in the wheat-growing period under limited irrigation.

Furthermore, N application reduced wheat yield in normal year (Figure 4), which is due to the production of a large number of ineffective tillers in the early stage (Cui et al., 2008), through consuming a large quantity of water and nutrients, thus resulting in a reduction of DMpa and yield. Another reason is excessive wheat population caused mutual shading of wheat in the late growth stage, reduction in effective tillering, and delay in heading and flowering of wheat, which ultimately leads to a decrease in yield (Zhang et al., 2018). Moreover, N application had no significant effect on wheat yield of SW in dry years (Figure 4), suggesting that the noneffective of N fertilizer under water-limiting conditions. In other words, under limited-irrigation conditions, soil water but not N is the most determining factor for wheat growth and yield (Olesen et al., 2000; Albrizio et al., 2010). However, N application increased wheat yield in MW in 2020/2021 but not in 2018/2019 (Table 2), which is mainly due to precipitation variation between the 2 years (Figure 1). Furthermore, the impact on grain yield by legume inclusion was greater in dry years than that in normal year across different N application rates, and the yield variation can be attributed to the precipitation variation among the 3 years, which contributed to different ear number in the 3 years (Table 2). Thus, these results further confirmed that the impact of legumes inclusion and N application is dependent on available soil water and precipitation under restricted irrigation.

Overall, our results suggest that legume inclusion is not always a suitable way to increase subsequent cereal yield in arid or semi-arid areas, because legume may consume more water thereby lowering pre-sowing soil water storage for following crops. Moreover, N application cannot promote wheat yield due to water-deficient under limited-irrigation conditions. In a further study, the optimized field managements and more drought-tolerant wheat cultivars could be explored under the limited-irrigation condition to improve wheat productivity.

## Conclusion

Our results demonstrate that including soybean within cereal crop rotation systems does not necessarily benefit subsequent wheat yield, since soybean depletes soil water storage available for subsequent wheat. By the time wheat reaches anthesis, these cascading effects in dry years can manifest in reduced ear number and dry matter accumulation, leading to yield losses. We also showed that N application promoted tiller production but decreased the rate of effective ear and yield of wheat in both rotation systems in normal year, but increased wheat yields under summer maize–winter wheat rotation system in dry year. Overall, our study indicates that available soil water rather than mineral N or N application is more influential in wheat development and yield in rotation systems with limited irrigation. Thus we suggest that future work consider optimizing irrigation water use (e.g., irrigation scheduling) in concert with genotypes with high water-use efficiency to cope with potential water shortages or a changing climate in the North China Plain.

## Data Availability Statement

The original contributions presented in the study are included in the article/**Supplementary Material**, further inquiries can be directed to the corresponding author.

## Author Contributions

JN: conceptualization, methodology, data curation, formal analysis, and writing—original draft. JZho, JZha, MH, and KL: review and editing. XW: data curation and review and editing. PW, SW, and LY: formal analysis and investigation. HZ: conceptualization and review and editing. YY: conceptualization, project administration, funding acquisition, and writing—review and editing. ZZ: project administration and funding acquisition. All authors contributed to the article and approved the submitted version.

## Funding

This study was financially supported by the National Natural Science Foundation of China (31901470) and the National Key Research and Development Program of China (2016YFD0300205-01).

## Conflict of Interest

The authors declare that the research was conducted in the absence of any commercial or financial relationships that could be construed as a potential conflict of interest.

## Publisher’s Note

All claims expressed in this article are solely those of the authors and do not necessarily represent those of their affiliated organizations, or those of the publisher, the editors and the reviewers. Any product that may be evaluated in this article, or claim that may be made by its manufacturer, is not guaranteed or endorsed by the publisher.
