# Supplementary material for: Soybean Crops Penalize Subsequent Wheat Yield During Drought in the North China Plain
Source: Front Plant Sci. 2022 Jun 28;13:947132. doi: 10.3389/fpls.2022.947132 (PMC9274277; doi:10.3389/fpls.2022.947132)
Supplement: Supplementary file 1 [file Table_1.docx]

**Supplementary materials:**

**Figure S1**

**Figure S1.** Soil water content between summer soybean — winter wheat (SW) and summer maize — winter wheat (MW) cropping systems after summer crops (a, b, c) and winter wheat (d, e, f) harvest in the three experimental years.

Values are means + standard errors (*n* = 15). *, *p* < 0.05; **, *p* < 0.01.
